# The Fangshan/Family-based Ischemic Stroke Study In China (FISSIC) protocol

**DOI:** 10.1186/1471-2350-8-60

**Published:** 2007-09-10

**Authors:** Xun Tang, Yonghua Hu, Dafang Chen, Siyan Zhan, Zongxin Zhang, Huidong Dou

**Affiliations:** 1 Department of Epidemiology & Biostatistics, Peking University School of Public Health, Beijing 100083, China; 2 Key Laboratory of Epidemiology, Ministry of Education, Beijing 100083, China; 3 Department of Neurology, the First Hospital of Fangshan District, Beijing 102400, China; 4 Department of Clinical Laboratory Medicine, the First Hospital of Fangshan District, Beijing 102400, China

## Abstract

**Background:**

The exact etiology of ischemic stroke remains unclear, because multiple genetic predispositions and environmental risk factors may be involved, and their interactions dictate the complexity. Family-based studies provide unique features in design, while they are currently underrepresented for studies of ischemic stroke in developing countries. The Fangshan/Family-based Ischemic Stroke Study In China (FISSIC) program aims to conduct a genetic pedigree study of ischemic stroke in rural communities of China.

**Methods/Design:**

The pedigrees of ischemic stroke with clear documentation are recruited by using the proband-initiated contact method, based on the stroke registry in hospital and communities. Blood samples and detailed information of pedigrees are collected through the health care network in the rural area, and prospective follow-up of the pedigrees cohort is scheduled. Complementary strategies of both family-based design and matched case-spousal control design are used, and comprehensive statistical methods will be implemented to ascertain potential complex genetic and environmental factors and their interactions as well.

**Discussion:**

This study is complementary to other genetic pedigree studies of ischemic stroke, such as the Siblings With Ischemic Stroke Study (SWISS), which are established in developed countries. We describe the protocol of this family-based genetic epidemiological study that may be used as a new practical guideline and research paradigm in developing countries and facilitate initiatives of stroke study for international collaborations.

## Background

Stroke is a major cause of disability and mortality for adults worldwide [1]. Epidemiological studies have suggested an increase in the incidence of stroke over past decades in China [2] and other developing countries [3], whilst the proportion of ischemic stroke in China is increasing [4]. Both family history and twin studies provide evidence for a strong genetic component of ischemic stroke [5,6], but the molecular basis for genetic background of common ischemic stroke remains unclear [7,8]. The ability to identify high-risk genetic factors could promote advances both in the discovery of novel pharmaceutical targets and in the development of genetic screening for public health.

Genetic linkage analysis is a powerful tool for identifying genetic predispositions for instance, the deCODE genetics group in Iceland has identified the novel STRK1 locus on chromosome 5q12 by studying 476 Icelandic patients with stroke within 179 extended pedigrees [9]. Another example is the Siblings With Ischemic Stroke Study (SWISS), which is an ongoing multi-centered genetic study in North America [10,11]. Genetic association analysis remains a useful and more popular tool for fine mapping of complex disease genes in regions of linkage. Numerous association studies using a candidate gene approach have been attempted for ischemic stroke, while the results are conflicting and far from conclusive [12]. This inconsistency is often ascribed to small samples with inadequate statistical power, phenotype definition, biological complexity, inter-population heterogeneity, environmental exposures, analytical method and population stratification. The effect of population stratification on the results of association analyses are potentially even more severe especially when small effects are studied in very large studies [13]. However, family-based design provides a useful complementary strategy because of its robustness to population admixture and stratification [14,15]. But as an elderly-onset disease, rare family-based genetic studies are available for ischemic stroke so far.

Several additional factors affect the study quality, with appropriate sample recruitment strategy, logical variant selection, minimum genotyping error, relevant data analysis, and valid interpretation all essential to generation of robust findings [16]. Consequently, questions of study design, implementation, statistical methods, and interpretation can be important. Herein we describe the protocol of a family-based genetic study for ischemic stroke in Chinese population, the Fangshan/Family-based Ischemic Stroke Study In China (FISSIC).

## Methods/Design

### Overview and objectives

The FISSIC program is a community-based and hospital-centered genetic epidemiological study of ischemic stroke. The study design has two components: first, a family-based study of ischemic stroke pedigrees, including probands, their siblings, and their parents; second, the traditional matched case-control study of ischemic stroke cases and their unaffected spouses. Cases with confirmed ischemic stroke are included as probands; after their informed consent is obtained, their parents, siblings, and unaffected spouses are recruited and screened by using the proband-initiated contact method [10]. Stroke status is verified at the central hospital (the First Hospital of Fangshan District, Beijing, China), and the index stroke for each case is subtyped by medical records. Baseline clinical and demographic data are collected by questionnaire, and longitudinal follow-up visits are scheduled. Blood samples are collected from all enrolled participants through the three-tier prevention and health care network (village, township and county level) in the study area. The samples are sent to the central laboratory for processing, testing, and genotyping. The genotype data are then merged with the clinical, environmental, and follow-up data for analysis. Comprehensive statistical methods are applied to both family-based and case-control data to ascertain potential complex genetic and environmental factors and their interactions.

The primary aim of the FISSIC is to study which genetic factors predispose to ischemic stroke, and the exploratory objectives are threefold:

1. To study predisposition of putative risk factor polymorphisms and their haplotypes in cases with different subtypes of ischemic stroke.

2. To investigate whether any association found between ischemic stroke and the panel of tested polymorphisms is influenced by sex, age, or smoking status and other environmental risk factors due to gene-environment interactions.

3. To detect and characterize the potential epistasis or gene-gene interactions between putative risk factor polymorphisms.

Understanding these objectives would be facilitated by having the following information collected in the FISSIC program: (1) DNA to document genetic predispositions, (2) environmental risk factor data obtained from the structured questionnaire, (3) serum indicators testing as intermediate phenotypes, and (4) regular follow-up clinical evaluations to document the occurrence of incident ischemic stroke.

### Study area selection

**Figure 1:**
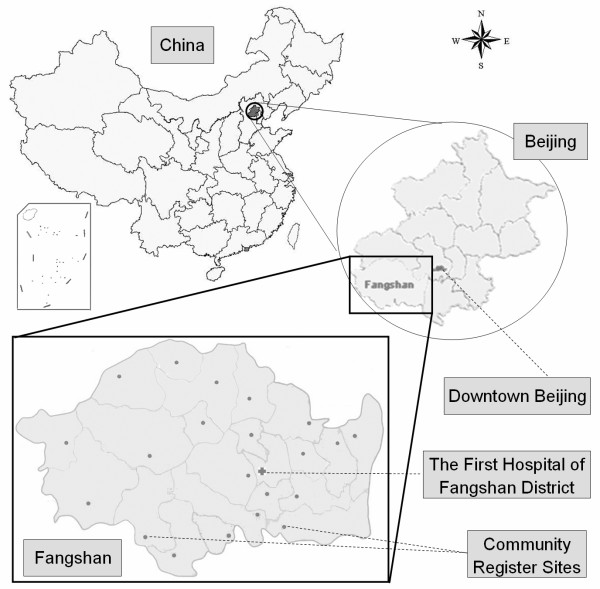
**Geographic map of the Fangshan/Family-based Ischemic Stroke Study In China (FISSIC) program region**. The cross symbol and spots symbols in the map of Fangshan indicate the Central Hospital (the First Hospital of Fangshan District) and Community Register Sites respectively.

The Fangshan District (39°30'~39°55' N, 115°25'~116°15' E), with an area of 1,866.7 square kilometers and a population of 760,000, is situated 45 kilometers southwest of downtown Beijing in China (Figure 1). The region, which is 60% mountainous, was selected because its population is representative of the rural northern Han Chinese. Furthermore, similar to the "stroke belt" in the south-eastern United States, this area is located in the "stroke belt" of China [17]. The First Hospital of Fangshan District is the central hospital in the study, and provides stroke unit for both primary and tertiary care to the rural population. Thus, sufficient cases have been proposed to enroll for the study.

### Participant eligibility

Four groups of subjects will be studied: ischemic stroke probands, their siblings, their parents, and unaffected spouses.

Probands will include all patients aged above 18 years, who are newly or have been ever referred to the First Hospital of Fangshan District with computerized tomography (CT) or magnetic resonance imaging (MRI) confirmed ischemic stroke. Stroke is defined according to the World Health Organization (WHO) criteria as commonly used [17]. Probands will not be excluded from the study for radiographic evidence of hemorrhagic transformation of an ischemic stroke. If probands have had more than one ischemic stroke, the most recent is the proband index stroke. Detailed inclusion and exclusion criteria for enrollment and recruitment are given in Table 1.

**Table 1:** Inclusion and exclusion criteria for probands in the FISSIC program

**a Inclusion criteria**
1. Diagnosis of at least one ischemic stroke confirmed by the study neurologist on the basis of history, medical records, and head imaging by CT or MRI;
2. At least 18 years old by the time of enrolment in the study;
3. At least one full sibling or parent alive in areas nearby;
4. Written informed consent by the patient or surrogate.
**b Exclusion criteria**
1. Diagnosis of TIA only;
2. Diagnosis of vasospasm after subarachnoid hemorrhage;
3. Diagnosis of some Mendelian disorders: CADASIL, Fabry disease, MELAS, or sickle cell anaemia;
4. Diagnosis of iatrogenic ischemic stroke associated with a surgical/interventional procedure such as coronary artery bypass grafting, carotid endarterectomy, or heart valve surgery;
5. Diagnosis of ischemic stroke associated with autoimmune condition or endocarditis.

Siblings are eligible for inclusion if they have a full sibling participating in the study as a proband, have also aged above 18 years at the time of enrollment, have provided written informed consent, and agreed to donate blood samples. Step siblings and adopted siblings are not eligible. Parents of the probands will be recruited whenever possible, but step parents are ineligible. All stroke-free spouses of probands are eligible to serve as additional controls.

### Recruitment procedures

**Figure 2:**
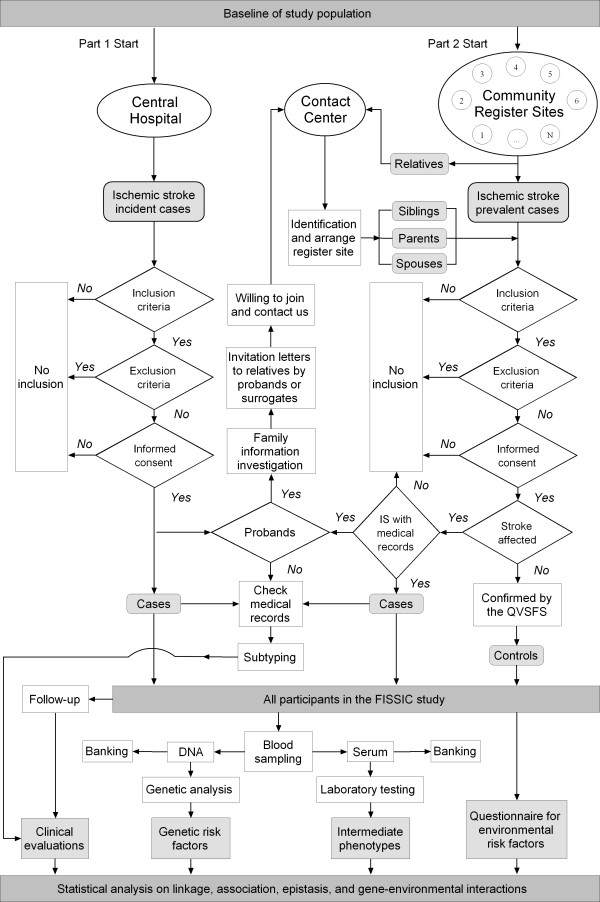
**Flow chart for recruitment of ischemic stroke probands and their relative controls in FISSIC program**. Abbreviations: FISSIC, the Fangshan/Family-based Ischemic Stroke Study In China; IS, ischemic stroke; QVSFS, the Questionnaire for Verifying Stroke-Free Status.

The FISSIC program procedure is summarized in Figure 2. Ischemic stroke probands will be enrolled in two parts: incident cases from the central hospital and prevalent cases from communities.

In part I, all patients with suspected stroke admitted to the neurology services at the central hospital are screened in the hospital-based stroke registry. A study neurologist will invite qualified patients to participate in a nontherapeutic stroke genetics study as probands, and then probands or their surrogates will take invitation letters to family members living nearby. The family members, who are interested in the study and willing to join, may call the Contact Center for details or register.

In part II, patients with a history of CT- or MRI-confirmed ischemic stroke, will be registered through the community-based health care networks in this rural area. With their permission, probands' medical records will be checked for qualification, and only after their family members also register in the study, will the next stage start. The Contact Center will not contact family members directly before they register.

All probands and their family members will be identified and arranged to any of the nearest community sites in the health care network or the Central Hospital with their own unique ID numbers. At the promised time and site convenient to the subjects, they will be evaluated by a study investigator according to a standard questionnaire and take the physical examination with informed consent, and then blood samples are collected by professional nurses. All the subjects will be informed about the study and agree for blood sampling and medical records reviewing by the study, thereafter written informed consent will be obtained.

### Data collection

All participants are interviewed in person by the study investigators, and will complete the following information:

#### Questionnaire

The structured questionnaire collects information on demographic characteristics (age, sex, education, etc), family history (stroke history of family members), medical history (age of first onset, diagnosis and treatment of stroke and other chronic diseases), environmental and lifestyle risk factors (smoking, drinking, physical activity, etc) and for women, history of menstruation and pregnancy.

According to the self-reported stroke history from the questionnaire by all participants in FISSIC program, detailed medical records will be checked retrospectively for positive reporting of stroke history to verify the diagnosis, and then will be labeled as "Cases"; the Questionnaire for Verifying Stroke-Free Status (QVSFS) [18,19] will be used for negative reporting of stroke history to avoid the bias of misclassification, negative answers for all items contained in the QVSFS will be labeled as "Controls"; Otherwise, the one with positive reporting but without medical records and the one with negative reporting while fail in screening of the QVSFS will be labeled as "Uncertainties", which are expected to be evaluated in the follow-up scheme.

#### Clinical evaluations

For each case of ischemic stroke, a study neurologist will check the medical records for evaluation, including patient history, physical examination, CT or MRI of the head, and laboratory testing. Where clinically indicated, the evaluation may also include magnetic resonance angiography, carotid ultrasonography, transcranial cerebral Doppler ultrasonography, color duplex flow imaging, resting and ambulatory electrocardiography, intracranial arterial imaging, and additional serum testing. Two different neurologists will subtype according to the Trial of Org 10172 in Acute Stroke Treatment (TOAST) criteria [20], and when the two neurologists disagree, the final decision will be made by the Stroke Verification Committee (SVC). This is a central specialist committee independent of the study neurologists, which adjudicates the diagnosis and subtype of ischemic stroke.

#### Physical examination

During the physical examination, anthropometric measurements will be obtained by trained and certified observers. Height will be measured without shoes by a fixed stadiometer and weight without heavy clothing by traditional scales. Body mass index is calculated as weight to height squared (kg/m^2^). Waist and hip circumferences are measured with patients standing relaxed and in light clothing. Waist circumference is measured horizontally at the midpoint between the lower costal margin and the iliac crest, and hip circumference at the level of maximum extension of the buttocks. Blood pressure will be measured three times at the right brachial artery by using a sphygmomanometer after the participant has been resting a seated position for 5 minutes.

#### Biochemical measures

Overnight fasting venous blood samples will be obtained to measure lipids, glucose, and other serum indicators. Blood specimens will be processed at the examination center and shipped to a central clinical laboratory in the Central Hospital for laboratory assays. Serum glucose, blood urea nitrogen, creatinine, concentrations of total cholesterol, triglycerides, low-density lipoprotein cholesterol, and high-density lipoprotein cholesterol will be measured by using the Hitachi 7180 autoanalyser (Hitachi High-Technologies Corp., Tokyo, Japan). Using the same autoanalyzer, serum apolipoprotein A-I, apolipoprotein B, apolipoprotein E, Lipoprotein (a) and high-sensitivity C-reactive protein will be determined by an immunoturbidimetric assay. Serum total homocysteine, folic acid, and vitamin B12 will be measured by immunoassay with the IMX Analyzer (Abbott Laboratories, USA). Additional blood samples will be frozen for future reference. Sera will be stored at -80°C, and blood clots will be stored at -20°C until DNA extraction.

#### Follow-up

The follow-up scheme will be developed for longitudinal assessments based on age and initial physical examinations. For example, subjects labeled as the "Uncertainties" or siblings with high blood pressure will be followed up most frequently. However, younger siblings will be followed up less frequently, every other year, depending on resources. The link between the baseline and the follow-up will be made by means of a unique ID number assigned to each participant. The purposes of follow-up are (1) to evaluate some subclinical stroke longitudinally and avoid the bias of misclassification, (2) to improve the recruitment of family members, and (3) to provide health education and promote healthy behavior.

### Selection of candidate genes and genotyping

Exhaustive genotyping for association in the human genome is impractical. Candidate genes for ischemic stroke are grouped into those for lipid metabolism, renin-angiotensin system, nitric oxide production, homocysteine metabolism, hemostasis, and other pathways [7]. Candidate genes in the first stage of the FISSIC program include genes for apolipoprotein E (*ApoE*), lipoprotein lipase (*LPL*), angiotensin-1-converting enzyme (*ACE*), endothelial nitric oxide synthase (*eNOS*), 5, 10-methylenetetrahydrofolate reductase (*MTHFR*), and plasminogen activator inhibitor 1 (*PAI-1*). These genes were selected because the gene product might relate to pathogenesis of disease. For example, hyperhomocysteinemia, atherosclerosis, and thromboembolism have been implicated in the development of ischemic stroke; however, hitherto results on associations between polymorphisms in these common genes and risk of ischemic stroke are conflicting [12]. Moreover, data in the Chinese population from the International HapMap Project [21] will facilitate the selection of tag-SNPs in these genes for haplotype analysis.

DNA from all subjects will be isolated from blood clots by phenol/chloroform extraction and ethanol precipitation, according to the standard procedure. Genotyping will be performed using the TaqMan fluorogenic 5' nuclease assay (Applied Biosystems). All PCR and end point fluorescent readings will be undertaken on an ABI PRISM 7900 HT sequence detection system (Applied Biosystems). Genotyping will be performed for the polymorphisms of interest, including *ApoE *ε4, *MTHFR *C677T, *eNOS *G894T, *PAI-1 *4G/5G, *ACE *I/D, and *LPL *S447X polymorphisms. In addition, several other polymorphisms may also be studied. The genotyper will be blinded to outcome and stroke subtype.

### Sample size and recruitment goals

We anticipate that the study sample will be mainly native Han Chinese from rural areas of northern China, and the baseline risk for ischemic stroke in this study population (P_0_) is 0.005; giving type I error (*α *= 0.05) and type II error (*β *= 0.20) for two-sided test, the sample size requirements for case-sibling, case-parent and matched case-control design of gene-gene interaction are dependent on the frequency of the polymorphisms of candidate genes, all designs provide an estimate of interaction on a multiplicative scale. The common polymorphisms of candidate genes we selected above have considerable allele frequency, for example, considering the allele frequency of 0.344 and 0.236 for *MTHFR *677C and *eNOS *894T respectively in this population from prior study, set 2.0 for the main gene effect (R_G_, R_H_), 3.0 for gene interaction effect (R_GH_) and dominant for mode of inheritance, the sample size requirements calculated by the QUANTO [22] program (Version 1.2.1) are 557 for case-sibling design, 321 for case-parent design and 418 for matched case-control design respectively.

Because ischemic stroke is a late-onset disease, and recruiting sufficient case-parents trios will be almost impossible, case-sibling pairs will be considered when calculating recruitment goals. Each inhabitants over 60 years old has average 3.5 siblings in this area in 2003, suppose every proband with two siblings consist 2 case-sibling pairs, nearly 280 pedigrees is needed. Considering missing data, genotyping error and stoke subtyping, sample sizes for recruitment goals of approximately 500 pedigrees. Recruitment began in June 2005 and is expected to continue until December 2010.

### Statistical methods

Because different types of designs are involved in the FISSIC program, including family-based design and case-control design, comprehensive statistical methods are under consideration. In general, for the family-based design with probands, siblings and parents, the Family Based Association Test (FBAT) method [23] will be used for association analysis. In addition, the Association in the Presence of Linkage (APL) test [24] is another family-based association method for nuclear families with multiple affected siblings and missing parental genotypes. For cases with affected siblings, linkage analysis will be performed by the affected sib pair (ASP) method, and discordant sib pairs (DSPs) will enhance the ASP linkage analysis to detect epistasis. For example, the Haseman-Elston (HE) regression method [25] may be used for the linkage analysis of quantitative traits. DSPs can also be compared as a matched case-control design with homogenous genetic background, while unaffected spouses can be compared as the matched case-control design with unrelated genetic background. Some conventional and novel approaches will be implemented in the case-control design for detecting associations and interactions for example, the logistic regression and the multifactor dimensionality reduction (MDR) method [26].

### Ethical aspects

This study was approved by the ethics committee of Peking University Health Sciences Center and each local institutional review board. Oral informed consent will be obtained from every participant before data collection, and written informed consent will be signed by every study participant or surrogate before blood sample collection. All participants will receive feedback reports and suggestions about their own health status under the privacy policy, and participants with untreated conditions identified during the examination will also be referred to a primary healthcare provider.

Because of the highly sensitive nature of genetic information, no individual information on genetic data will be informed. Each investigator who obtains or has access to individual personal identifiers is blinded to genotypic data. As commonly addressed in genetic studies [27], the following rules will be obeyed: Subjects may consent to or refrain from participation in future research at the time of the initial consent process. They may declare future-use agreement to join the original study only or any study, and applications for future use will be reviewed formally.

## Discussion

Ischemic stroke is a complex function of multiple genetic and environmental risk factors. Family-based genetic study of ischemic stroke can be used to perform linkage and linkage disequilibrium mapping in pedigrees, and compared with the standard case-control design, family-based design has the advantage of robustness to population stratification. Some significant findings of predispositions such as the gene encoding phosphodiesterase 4D (*PDE4D*) [28], may vary between populations and the replications in different studies will provide more powerful evidences [29]. Unfortunately, family-based ischemic stroke study from Asia, especially China, is currently underrepresented.

Genetic studies in isolated populations, such as the deCOAD study in Iceland [9], are theoretically robust, but they require an integrated health care database not available in many countries. Although stroke registries have been established successfully in Western countries, only a few hospital-based stroke registries have been established in some cities of China until recently [30]. Multi-centered study design, like that of the SWISS study [10] in the United States, may not be appropriate in China because of quality control and logistic difficulties at the present stage. Recently a new pedigree study of stroke in Sweden has been reported [31], and their stroke pedigrees were ascertained retrospectively from a population-based stroke registry at the northern Sweden MONICA (MONItoring of trends and determinants in CArdiovascular disease) Center. Similarly, the SINO-MONICA project [17] facilitated epidemiological studies of cardiovascular disease in some areas of China. The FISSIC study has been inspired by these studies, but the design differs in many perspectives.

The Fangshan District is a rural mountainous area with a history of low immigration; family members tend to stay congregated, consequently genetic diversity in the northern Han Chinese population is relatively low and pedigree recruitment is convenient. Epidemiological studies of chronic diseases in this area have long traditions, for instance the Asia Pacific Cohort Studies Collaboration (APCSC) [32], many local health care staff has been trained and some baseline data are available, and meanwhile the public has a favorable attitude towards genetic research and community consent is probably possible. Most concerning, however, is the high prevalence of stroke and hypertension among adults in Fangshan District. Most inhabitants will be hospitalized for stroke emergency in the First Hospital of Fangshan District within 6 hours after onset. This hospital keeps detailed medical records on incident cases and most prevalent cases from the communities.

We propose to initially register approximately 500 pedigrees in five years. Preliminary pilot study from two communities in Fangshan District showed that the project is feasible [33]. In the parallel case-control study, unaffected spouses will serve as matched controls. The use of spousal controls is convenient and can help control for adult environmental exposure. Spouses can be expected to have spent a large proportion of adulthoods with the patients, and they likely have shared similar environmental exposures, e.g., diet or exposure to cigarette smoke. Spouses will also facilitate pedigree recruitment as surrogates of the probands.

Unaffected controls, especially young siblings, may have subclinical disease, which could lead to the bias of misclassification. In addition to using the QVSFS [18] for validation, longitudinal follow-up will provide a prospective element to the study. An annual physical examination and other phenotype data collection will be scheduled for up to five years in order to investigate changes in stroke-related phenotypes. This follow-up design allows extending the pedigree recruitment to more potentially affected siblings and even offspring in the future.

For environmental exposures not easy to measure by questionnaire, serum biochemical indicators will be tested as intermediate phenotypes. For example, homocysteine is an important risk factor for ischemic stroke [34]; therefore, we will study serum homocysteine and regard serum folic acid and vitamin B12 level as surrogate indexes for the environmental factors (vegetable intakes). As another marker for early carotid atherosclerosis, the carotid artery intimal medial wall thickness (IMT) by ultrasonography will be proposed for later study as an intermediate phenotype for large vessel ischemic stroke subtype. Intermediate phenotypes represent specific components of the disease process and can be expressed as continuous variables for quantitative trait analysis, rather than the presence or absence of disease itself [35].

The complexity of ischemic stroke reflects the contribution of polygenic effects to disease process and the interactions of these multiple genes with a multitude of environmental factors. Many common genetic factors individually play little role in the development of ischemic stroke [8]. However, specific polygenetic patterns, alone or in combination with other common environmental factors, can exert a synergistic effect on the evolution of a specific ischemic stroke subtype. For instance, synergistic effects in ischemic stroke between the *ACE *D/D and *MTHFR *677TT genotypes and alcohol drinking or smoking have been reported by Szolnoki et al [36]. New advances in the study design and statistical analysis of family-based studies have expanded the range of hypotheses that can be tested, including gene-gene and gene-environment interactions in ischemic stroke. In the FISSIC program, linkage and family-based association analyses are applied simultaneously to maximize use of family data sets, and gene-environment and gene-gene interactions will be tested by comprehensive methods as well. Each of these methods offers advantages in different situations, e.g. the FBAT method is designed to find loci with main effects instead of interactions, while the MDR approach is useful in identifying gene-gene interactions among minor genes [37]. This combined strategy will help us discern a true association from a spurious finding.

Some limitations of the FISSIC should be mentioned: the National Institutes of Health Stroke Scale (NIHSS) is not used to classify stroke by severity, no genome-wide linkage screen is undertaken and no cell lines are created, for lack of technical feasibility at the current stage in developing countries. Although we recognize the value for genome-wide linkage screen, we will undertake it at a later stage or bank DNA samples for future research.

Recently the launch of new NCBI database of Genotype and Phenotype (dbGaP) addresses the critical need for sharing of genotype and phenotype information, and thousands of SNPs have been released through collaborations such as the SNP Consortium [38]. Hence, any potential collaboration proposals are welcome and will be evaluated by investigators in the FISSIC program to make full use of the resources available.

In conclusion, the FISSIC program is a family-based genetic study of ischemic stroke, focusing on the genetic and environmental determinants and their interactions with comprehensive strategies. Collecting DNA samples from a large cohort of ischemic stroke pedigrees in a Chinese population is really a promising challenge rather than mission impossible. A long-term prospective follow-up of the pedigree cohort will offer unique resources for future genetic research on ischemic stroke. The FISSIC is expected to be a new opportunity to identify stroke predisposition genes and facilitate initiatives of stroke study for international collaborations.

## Abbreviations

APCSC, Asia Pacific Cohort Studies Collaboration; APL, Association in the Presence of Linkage test; ASP, Affected Sib Pair; CADASIL, Cerebral Autosomal Dominant Arteriopathy with Subcortical Infarcts and Leukoencephalopathy; CT, Computerized Tomography; DSP, Discordant Sib Pair; FBAT, Family Based Association Test; FISSIC, Fangshan/Family-based Ischemic Stroke Study In China; MDR, Multifactor Dimensionality Reduction; MELAS, Mitochondrial Encephalomyopathy, Lactic Acidosis, and Stroke-like episodes; MONICA, MONItoring of trends and determinants in CArdiovascular disease; MRI, Magnetic Resonance Imaging; NCBI, National Center for Biotechnology Information; NIHSS, National Institutes of Health Stroke Scale; QVSFS, Questionnaire for Verifying Stroke-Free Status; SNP, Single Nucleotide Polymorphism; SVC, Stroke Verification Committee; SWISS, Siblings With Ischemic Stroke Study; TOAST, Trial of Org 10172 in Acute Stroke Treatment; WHO, World Health Organization.

## Competing interests

The author(s) declare that they have no competing interests.

## Authors' contributions

XT drafted the manuscript, participated in the design and coordination of the study, and will perform statistical analysis of the data. YH conceived of the study, participated in its design and coordination, and finalized the manuscript as the principal investigator of this study. DC participated in the design of the study, and is responsible for blood sample storage and genotyping. SZ participated in the design of the study, and is responsible for coordination and quality control of the study. ZZ participated in the design of the study, and is responsible for recruitment and clinical assessment. HD participated in the design of the study, and is responsible for blood sample processing and clinical laboratory assays. All authors contributed to the writing of the study protocol in an iterative manner, and have read and approved the final manuscript.

## Pre-publication history

The pre-publication history for this paper can be accessed here:


